# Re-examining Lopes's computationalist interpretation of Husserlian phenomenology: a critical perspective

**DOI:** 10.3389/fpsyg.2026.1903168

**Published:** 2026-08-27

**Authors:** Chengcheng Wu

**Affiliations:** Institute of Humanities and Arts, Shandong University, Qindao, China

**Keywords:** computational theory of mind, Fodor (Jerry), Husserl, logic of signs, parallel structure

## Abstract

This paper critically examines the view that Husserl's 1891 essay *The Logic of Signs (LoS)* anticipates the Computational Theory of Mind (CTM). According to this interpretation, Husserl's account of symbolic inference endorses an automatic, syntactic, causally driven model of thought. However, this study argues that such a reading neglects Husserl's broader philosophical context and his explicit rejection of early computationalist views. By situating LoS within Husserl's evolving theory of signs and tracing its development through *Logical Investigations* and later works, the paper shows that Husserl's “parallel structure” of psychological mechanisms and logical forms serves to justify the legitimacy of symbolic thinking, not to reduce cognition to computation. The analysis identifies key mismatches between CTM and Husserl's framework, including differences in scope, structure, and the role of semantics. It concludes that LoS offers a provisional, non-committal answer to the “logic problem of signs” and that interpreting Husserl as a computationalist risks misrepresenting his project. A more accurate approach recognizes Husserl's openness to multiple forms of cognition and emphasizes the normative, a priori laws governing different modes of thought.

## 1 Introduction

Lopes (2020) reads Husserl's 1891 essay *The Logic of Signs (semiotic)* (*LoS*) as an early endorsement of the Computational Theory of Mind (CTM).^1^ According to CTM that refereed by Lopes, the mind is an automatic, formal system that manipulates syntactic symbols through causal mechanisms; Lopes argues that Husserl's description of the “natural psychological mechanism of symbolic inference” (Husserl, 1970, p. 363; Husserl, 1994, p. 42) in *LoS* fits this model (shows as Table 1).

**Table 1:** Summary of Lopes's argument.

CTM		***LoS***	
Single-line structure		Dual-line structure	
**The mind is an automatic**		**Symbolic thinking is an automatic**	
		Psychological part (mechanical procedure 1)	Logical part (mechanical procedure 2)
**Symbol-manipulating**	Symbols are semantically evaluable	**Symbol-manipulating**	Authentic judging: here exist *X*(*A, x*1, *x*2…), Y(*A, y*1, *y*2…), if a judgment about *A* holds in *X*, then it also holds in *Y*.
	Symbols are univocal		Inauthentic judging: (1) premise implies conclusion; (2) symbols are univocal.
**Formal** syntax		**Formal** psychological laws	Logical laws
**Driven by causality**		**Driven by causality**	Driven by knowledge (Erkenntnismotiv)
**Preserving truth system**		**Preserving truth system**	**Producing assured truth system**

This table presents Lopes's summary of CTM (on the left side) and his summary of Husserl's LoS (on the right side). The only part I added to the table based on Husserl's text is “producing assured truth system” in the lower-right cell, in order to complete the parallel between the two mechanical procedures.

However, this interpretation remains problematic unless LoS is understood within its full philosophical context. I examine whether this selective reading of Husserl aligns with the broader framework of his theory of signs. A textual clue warns us to approach Husserl's computational stance cautiously, as he explicitly rejects a certain computational view of the mind. While Lopes rightly links Husserl's work to the truth-preserving problem central to CTM, he misinterprets Husserl's notion of parallel structure by reducing it to CTM assumptions. In conclusion, a neutral interpretive stance remains most defensible, as it is still unclear whether Husserl truly endorsed a CTM-like position.

## 2 A clue: Husserl's opposition to “computational mind”

An appendix in *Husserliana* XXII, written in 1891—the same year as *LoS*—and titled *From Husserl's Sketches for His Review of Schröder*, presents Husserl's rejection of the computationalist view represented by Hobbes:

*Schröder* speaks as if, with the introduction of a symbolic language in place of the natural language of words, the algorithmic procedure were already given. It is this same confusion which we already find in *Leibniz—*and in fact even, after a fashion, in *Hobbes*. Thinking is a type of calculating (Denken ist eine Art des Rechnens), the eccentric philosopher last-named supposes. Expressions in terms of symbols are essential to thinking. Judging is realized with and in linguistic signs. But language utilizes signs in many ways, as the special language of arithmetic utilizes the number signs. And in both cases the combination of the signs corresponds to the combination of thoughts—or rather the two are one, as, in the particular thought, external sign and interior correlate are one. Thus, for him, speaking and calculating coincide (Husserl, 1979, p. 389/1994, p. 430–431).

This passage underscores Husserl's distance from early computationalism: he rejects equating the mind with computation and warns against conflating symbol manipulation with genuine cognition of the objects represented. Such conflation leads, as with Hobbes, to reducing thinking to calculation and thus misunderstanding the role of symbols. This clue cautions against reading Husserl's early theory of signs as computationalist. Lopes's argument, based on a single text, likely misreads Husserl. To argue that *LoS* truly depicts a computationalist view of mind, one must first understand the tasks described there. In fact, the “logic of signs” in this early work can only be fully grasped through contemporaneous and later writings.

## 3 Clarifying “the logic problem of signs” in *LoS*

What is the “logic problem of signs” that Husserl raises in *LoS*? Its origin lies in his study of arithmetical concepts. In calculation, numbers serve merely as signs standing in for the concepts of number themselves. For example, the figure “1” is not the same as the concept “one” it represents. Yet when applying algorithms, we operate solely on mathematical symbols; as long as these operations follow the algorithm's rules, the results are guaranteed correct. Husserl noted that this substitutive use of signs lacked a proper logical justification—logicians at that time had not explained why it works. As he wrote in an 1890 letter to Stumpf:

In recent decades, the number has repeatedly been defined as a sign: does arithmetic then deal merely with signs? Would it therefore be nothing more than a mere game of signs? Why not, says Helmholtz, if the game only has the great advantage of allowing all these “applications”? But who explains to us how a mere game of signs can allow applications at all? Helmholtz has not done this in the slightest. He falls into the same error as his predecessors: numbers (or ordinal numbers) are regarded as the foundation of arithmetic, yet—even when taken merely as signs—they are explained through the sequence of number-signs. Since every sensible person realizes that such profound concepts cannot simply be conjured away by means of notations, how could one possibly approve of such a theory of arithmetic? (Schuhmann, 1977, p. 158).

Husserl then thought that new logic must solve the logic problem of signs:

An algorithm is, therefore, a systematic method of symbolic inference; a language, a systematic method of expression of thoughts and psychical phenomena in general by means of signs. How it is possible that a blind mechanism of sensible symbols can replace and spare us logical thinking? That is the great problem for the logic of signs, for semiotics…… A deductive logic without a semiotic appears to me not to deserve its name at all. The reproach which this involves falls upon the whole of logic, of the present and the past; but in a heavier degree upon symbolic logic, since it itself regards the development of symbolic methods as its chief aim (Husserl, 1979, p.394/1994, p. 436).

In the texts discussed above, Husserl distinguishes two types of sign systems: linguistic and arithmetical. While they share certain similarities, their differences are more significant. Nevertheless, both face the same fundamental issue: the logical legitimacy of such systems. In his later reflections on symbolic thinking, Husserl repeatedly emphasized their “blind” character. For example, in arithmetic, the transition from numerical signs to the concepts they represent occurs in a blind, direct manner; similarly, in language, the shift from words to their meanings is likewise blind and immediate (see Bennett, 1988, p. 17). Husserl therefore argued that logic must take on a new task: to explain how a form of thinking marked by blindness can nevertheless yield correct results. This is what he called the “logic problem of signs.”

Against this background, Husserl's aims in *LoS* can be summarized as: (1) classifying signs by their substitutive function; (2) offering a psychological account of the mechanisms behind symbolic thinking (its blind, causal character); and (3) providing a logical justification of symbolic thought (logical forms paralleling the psychological one). In *LoS*, Husserl intertwines the latter two tasks. By this stage, he had already distinguished between arithmetical and linguistic sign systems (Husserl, 1979, p. 394/1994, p. 436), yet his central concern remained the legitimacy of all sign behavior. To unify these systems, he introduced a new distinction: authentic/inauthentic judgments (Husserl, 1970, p. 359/1994, p. 38).^2^ Constructing the logical forms of these two types of judgment, he argued, would resolve the question of legitimacy. Based on whether signs allow direct access to their corresponding concepts, all sign behavior can be subsumed under this framework. Husserl sought to show that our blind symbolic mechanism is governed by a parallel logical form operating unconsciously during symbol use. The task of symbolic logic, therefore, is to uncover and explicate these forms. He sketched the logical forms of both judgment types and described their features to argue for the logical legitimacy of symbolic thinking (see Table 1 for the parallel structure).

## 4 Did Husserl maintain his early answer to “the logic problem of signs”?

In fact, the two types of logical forms of judgment that Husserl proposed in *LoS* face several challenges. One relevant issue concerns the definition of the logical form of inauthentic judgments, especially in relation to the logical construction of arithmetic signs. In the use of arithmetic symbols, there exists a threefold distinction: between number concepts themselves, numerical symbols, and general (or schematic) symbols. For example, the commutative law in mathematics is usually expressed as *a* + *b* = *b* + *a*, where *a* and *b* can stand for any number. A concrete instance like *1* + *3* = *3* + *1* uses the numerals “1” and “3,” which are themselves number symbols, distinct from the number concepts they represent. These three layers are not strictly parallel: substitution between general symbols and number symbols is arbitrary, and even substitution between number symbols and number concepts can sometimes be arbitrary. Thus, Husserl's requirement for univocity in the logical forms he constructed here faces serious difficulties.

Furthermore, the second feature of these logical forms, their satisfaction of purely formal requirements, applies only to the logic of arithmetic algorithms. Linguistic symbol systems involve entirely different logical form requirements. As Husserl came to a deeper understanding of linguistic signs, he gradually abandoned the simplistic approach found in *LoS*—that is, assigning fixed logical forms to each type of sign behavior. In the work written three yeas later, Husserl suggested that the problem of semiotics must be addressed through an essential analysis of intuitive and representational acts.

Now I certainly will not deny that one can considerably advance logical understanding of the soundness of symbolic thought (and above all, of course, mathematical thought) without a more penetrating insight into the essence of those elementary processes of intuition and the representation which everywhere make that thought possible. But without such insight one surely cannot obtain a full and truly satisfactory understanding of symbolic thought or of any logical process (Husserl, 1979, p. 122/1994, p. 168–169).

From the above, it is clear that any investigation into symbolic behavior, once it examines the differences between various types of symbols in depth, must account for a wider range of possible variations, which Husserl eventually recognized. Münch notes that the core of this shift lay in Husserl's recognition of the expressive function of linguistic signs:

The issue is that, according to *Philosophy of Arithmetic*, the use of symbols is always blind, and, thus symbolic cognition is absurd. Although the goal of clarification was to construct algorithms parallel to natural processes, Husserl merely described the characteristics of these algorithms by loosely referencing the user's cognitive motivations. It was only upon realizing that language also has an expressive function, and that meaning (Meinen) is part of the structure of consciousness, that Husserl came to see the decisive task of the philosophy of logic as the analysis of meaning-intending consciousness, rather than justifying natural cognitive processes through the construction of parallel algorithms (Münch, 1993, p. 233).

Therefore, Husserl did not retain the rough solution to the logic problem of signs that he proposed in *LoS*. The brief answer given there was not his final position.

## 5 Why it is wrong to say Husserl had the computational theory of mind

From the supplementary materials above, we can identify Husserl's actual motivation for writing this article: to address the source of the logical legitimacy of blind inferences made through the substitution of symbols for conceptual objects. This is contrary to Lopes's claim that “Husserl's 1891 text derives from his desire to find a solution to the psychosemantic problem of how meaning is formally inferred by a causal mechanism” (Lopes, 2020, p. 29). At this stage, Husserl had not yet addressed the meaning of linguistic signs; only during the *Logical Investigations* did he develop a more comprehensive account. One might object that, setting aside Husserl's later work, the provisional solution he offers in *LoS* already makes him, in effect, a disguised proponent of CTM. This is a fair objection. Nevertheless, even if we confine our analysis to that paper alone, I would argue that Husserl's proposal still does not fully align with CTM.

At first glance, *LoS* appears to contain the elements needed for the CTM model Lopes outlines: a formalized, causally driven, symbol-manipulating system. Yet, this similarity is superficial. It is unconvincing to claim Husserl endorsed a computational theory of mind. Lopes's reading faces multiple problems, including misinterpretations of Husserl's concept of thinking, mismatches between linear and parallel structures, and a misreading of the role of formal rules. All of these undermine the computationalist interpretation.

First, Lopes conflates human symbolic thinking with human thinking as a computation-based symbolic system. As earlier evidence shows, Husserl consistently rejected the idea that thinking is identical to computation. Even when symbolic acts proceed without awareness of the concepts they express, the concepts themselves are not reducible to symbol manipulation. The “logical legitimacy of signs” arises precisely from distinguishing the two. In *LoS*, Husserl discusses symbolic behavior, primarily arithmetic, understood as a blind formal system. His aim is to explain the legitimacy of such symbols in relation to the concepts they substitute for, grounding that legitimacy in logical form. For Husserl, the mind includes many non-symbolic acts (e.g., perception) where the problem of sign legitimacy does not arise. CTM, by contrast, reduces all cognitive acts—whether intuitive, arithmetic, or linguistic—to a causally driven formal system. Lopes's reading therefore conflates symbolic thinking (a subset of thought) with a comprehensive computational model of the mind.

Second, Lopes's linear structure does not align with Husserl's parallel structure. The central question is which of Husserl's two parallel mechanical procedures, mechanical procedure 1 or mechanical procedure 2, as illustrated in Table 1, corresponds to the mechanical system of CTM as characterized by Lopes. On the one hand, it seems to correspond to mechanical procedure 1, since this procedure is causally driven. But if that is the case, then we cannot find any stipulated principles governing symbols along this line. On the other hand, Lopes consistently maintains that both CTM and Husserl are concerned with how a blind process can preserve truth. If we take this claim seriously, then CTM would have to correspond to mechanical procedure 2, for only this procedure yields assured truth. However, on the logical line, although the account does satisfy the rules governing symbols, the logical system is not driven by causality; rather, its discovery and construction are motivated by cognitive interests and realized through reflection (Husserl, 1970, p. 365/1994, p. 44). Even if we take Husserl's parallel structure here as a unified system, the forces driving the system are dual in nature: causal motivation and epistemic motivation. This diverges significantly from CTM, which posits a purely causally driven model. Even if we relax the requirement that CTM's syntactic rules match symbol-manipulation rules exactly, the fact remains that one type of CTM rule would have to correspond to two distinct types in *LoS*.

Thus, although Husserl's *LoS* contains elements Lopes associates with CTM, any strict equivalence between the two faces unresolved and substantial difficulties.

## 6 Where can we find the answer to the logic problem of signs: a proper understanding of the parallel structure

Lopes's interpretation rests on a misreading. He misunderstands both Husserl's motivation for this article and the purpose of the parallel system Husserl devised. Viewed from the standpoint of Husserl's later theoretical developments, the *LoS* answer to the logic problem of signs was provisional and simple. As his analysis of consciousness deepened, a more comprehensive answer appeared in *Logical Investigations*, and the logical issues first raised in *LoS* were only fully resolved in *Formal and Transcendental Logic*. We can thus clarify the problems mentioned in *LoS* by reading them through the lens of *Logical Investigations*, which also shows why Husserl's account of symbolic thinking cannot be equated with CTM.

In the *Introduction* to *Logical Investigations*, Husserl aimed to establish a general theory of a formal deductive system. With the development of his phenomenology, the inquiry into the pure laws governing acts of cognition became integral to this project. The logic problem of signs raised in *LoS* should thus be understood as the question: “What a priori logical laws regulate sign-acts?” or “What logical laws ground their legitimacy?” At this stage, however, Husserl had not fully freed himself from psychologism. He neither clarified the ideal nature of the “logical laws” he referred to nor examined the pure laws of the validity of meaning in signititive acts, leaving his *LoS* answer abrupt and preliminary.

Reconsidering the blind mechanism of signs in light of *Logical Investigations* brings greater clarity. In his phenomenological study of objectivating acts, the signitive acts (judgment acts) of *LoS* fall under knowing acts (categorical acts): “Acts of straightforward intuitions we called ‘sensuous'; founded acts, whether leading back immediately or mediately to sense, we called ‘categorial”' (Husserl, 1984, p. 712/2001, p. 306). Acts directly traceable to sensory intuition are “authentic thinking,” while those only indirectly traceable, or not traceable at all, are “inauthentic thinking”, corresponding to “authentic judgments” and “inauthentic judgments.” In *LoS*, Husserl observes that signs in authentic judgments can be traced directly to the sensory, whereas in inauthentic judgments they can only be traced indirectly, if at all. In *Logical Investigations*, he further shows that signs in inauthentic judgments, especially linguistic signs, must be connected to the sensory through meaning, while certain arithmetical signs resist such intuitive fulfillment.

Both authentic and inauthentic thinking are governed by a priori laws. Authentic thinking is regulated by “the laws, that is, of categorial intuitions in virtue of their purely categorial forms” (Husserl, 1984, p. 720/2001, p. 311), which can be seen as the mature form of the logical laws of authentic judgment mentioned in *LoS*. Inauthentic thinking is more complex. Using linguistic signs as an example, Husserl explains that grammatical laws regulate the signs, while the “pure laws of validity of meaning” regulate their meanings, which in turn refer to intentional objects. He regards these two sets of laws as strictly parallel and insists on the univocity of signs: “the ideal of a logically adequate language is that of a language which can give unambiguous expression to all possible matters and all possible categorial forms. To its words certain significative intentions unambiguously pertain, which can come alive even in the absence of ‘corresponding,' that is, of fulfilling, intuition. There is therefore, running parallel to all possible primary and founded intuitions, a system of primary and founded meanings which could possibly express them” (Husserl, 1984, p. 721/2001, p. 311). Husserl here points out that an ideal language is one in which signs univocally express meanings, and meanings univocally express (authentic) thinking.

Thus, the two logical laws of inauthentic judgment in *LoS* can be read as early versions of those later articulated for inauthentic thinking in *Logical Investigations*. In *LoS*, Husserl had not yet considered meaning as the mediating link between sign and thought, nor incorporated the ideal laws of meaning into logic. His early answer is therefore vague, but the work can be seen as a preview of his later research agenda. The parallel structure already distinguishes the task of objective logic within pure logic from that of epistemology within phenomenology. Full understanding of the sign system described in *LoS* thus requires the perspective of his later writings.

Let us return to the original question—Lopes's claim that both CTM and Husserl share a concern with the problem of “preserving truth” (Lopes, 2020, p. 26). As discussed in Part 3, Husserl was dissatisfied that the symbol system was nonetheless treated as the foundation of scientific knowledge without any logical inquiry into its legitimacy. This dissatisfaction drove his effort to reform logic, which he called “universal mathematics” (*Allgemeine Mathematik*, Schuhmann, 1977, p. 161). The starting point of this reform was precisely to address the logical problem of signs: to determine the basis on which a blind mechanism could serve as a valid means of producing scientific knowledge. Lopes interprets this as Husserl's answer to the question why can a blindly mechanism produce truth and reads Husserl's concern as the claim that, although the mechanism is blind, a parallel logical process must be posited—much like Fodor's computational metaphor, in which the mind is conceived as a syntax-driven machine (Lopes, 2020, p. 38).

However, as the preceding analysis shows, Husserl's logical side of the parallel structure is not metaphorical. It should be understood as an early attempt to identify the *a priori* laws governing objectifying acts—rules that normatively govern symbolic systems. Lopes claims that “Husserlian mechanism bears the properties of an automatic formal system, defined as a system whose formally designated, syntactic tokens are manipulated without recognition of their semantic denotations” (Lopes, 2020, p. 26). While Husserl does describe an automatic symbolic system, he does not disregard semantic reference; rather, he had not yet clarified the specific mode by which it operates within the system. Husserl repeatedly stressed that only by determining how semantic reference functions within a symbolic system can one fully justify its logical legitimacy. It must be acknowledged that Husserl's sustained search for essences and laws in his studies on signs can give the impression that he was proposing a formalized, mechanical conception of mind (or thinking). However, equating the sign system in *LoS* with Fodor's computational theory of mind faces serious difficulties. First, Husserl had already voiced objections to similar computationalist ideas in writings from the same period. Second, relying on a single text may risks misrepresenting the task Husserl pursued in *LoS*; mere overlap in isolated elements cannot establish theoretical equivalence between the two positions.

## 7 Conclusion

In this regard, Hubert Dreyfus's view remains persuasive: whether Husserl held a computational theory of mind is an open question (Dreyfus, 1982, p. 10). Even if this comparison opens a new interpretive path for understanding Husserl's phenomenology, the most faithful approach to his texts requires situating them in their full historical context and accurately grasping his motivations. Indeed, a critique of Lopes's argument clarifies Husserl's early writings, revealing that many central concerns later developed in *Logical Investigations* already appear in embryonic form in these works.

Considering current developments in artificial intelligence—especially large language models—philosophical views that closely link thinking to language are under increasing pressure. Deep learning neural networks manipulate language in ways that appear, in some respects, to transcend both syntactic and semantic rule constraints. These advances prompt renewed questioning of whether Fodor's computational conception of mind remains viable, and whether Husserl's investigation into the *a priori* rules of symbolic, especially linguistic, behavior retains its force in light of such changes. From the preceding analysis, a more reasonable position is that Husserl never proposed a fixed model of thought. His clearest claim is that different modes of thinking operate under different normative logical forms. Thought can proceed via symbols and algorithmic rules, but it can also directly engage with objects of knowledge or follow other cognitive paths. The defining feature of thinking, in Husserl's view, is its openness to multiple forms of cognition.

Thus, if we interpret Husserl through the lens of contemporary concerns, then in the present context of rapidly advancing computational technologies, arguing for a fundamental alignment between Fodor and Husserl on the theory of mind is unlikely to be the most productive approach. A more promising line of inquiry would examine whether their respective accounts of language and signs remain coherent and compelling within the emerging paradigm of large language models.

## Footnotes

1 Coincidentally, Dieter Münch also based on same article to argue that Husserl has the same idea with the physical symbol system hypothesis (PSSH) that formulated by Allen Newell and Herbert A. Simon, but very different with Fodor (Münch, 1993, p. 221–233).

2 If we refer to the original German edition, we can find the English translater make a small mistake on this part. On authentic judging, “the sigs ‘X' stands for our representation-possibly a phantasm in which the property X is exclusively attended to and judge of-the remainder being wholly obscure.” Here, “property X” should be “property A”. (Husserl, 1979, p. 360/1994, p. 39).
